# Aberrations in Angiogenic Signaling and MYC Amplifications are Distinguishing Features of Angiosarcoma

**DOI:** 10.4172/2329-9495.1000102

**Published:** 2013-04-08

**Authors:** Vittal Kurisetty, Brad A Bryan

**Affiliations:** Department of Biomedical Sciences, Paul L. Foster School of Medicine, Texas Tech University Health Sciences Center, El Paso, Texas, USA

**Keywords:** Angiosarcoma, Vascular tumor, Sarcoma, Genomics, MYC, Angiogenesis, Vascular endothelial growth factor, Hemangiosarcoma, Lymphangiosarcoma

## Abstract

Angiosarcomas are very aggressive, rare malignant tumors that originate from vascular or lymphatic vessels and primarily occur following chemical exposure or radiation therapy. Tumor response to either chemotherapy, radiation, or novel anti-angiogenic therapeutics is very low, and because little is known regarding the aberrant signaling that controls these tumors, personalized treatment options for many of these patients are lacking. In this review, we summarize several recent findings regarding the genomics of angiosarcomas, including new discoveries regarding aberrant angiogenic signaling and Myc amplification as key features of this tumor type.

## Introduction

**Figure 1:**
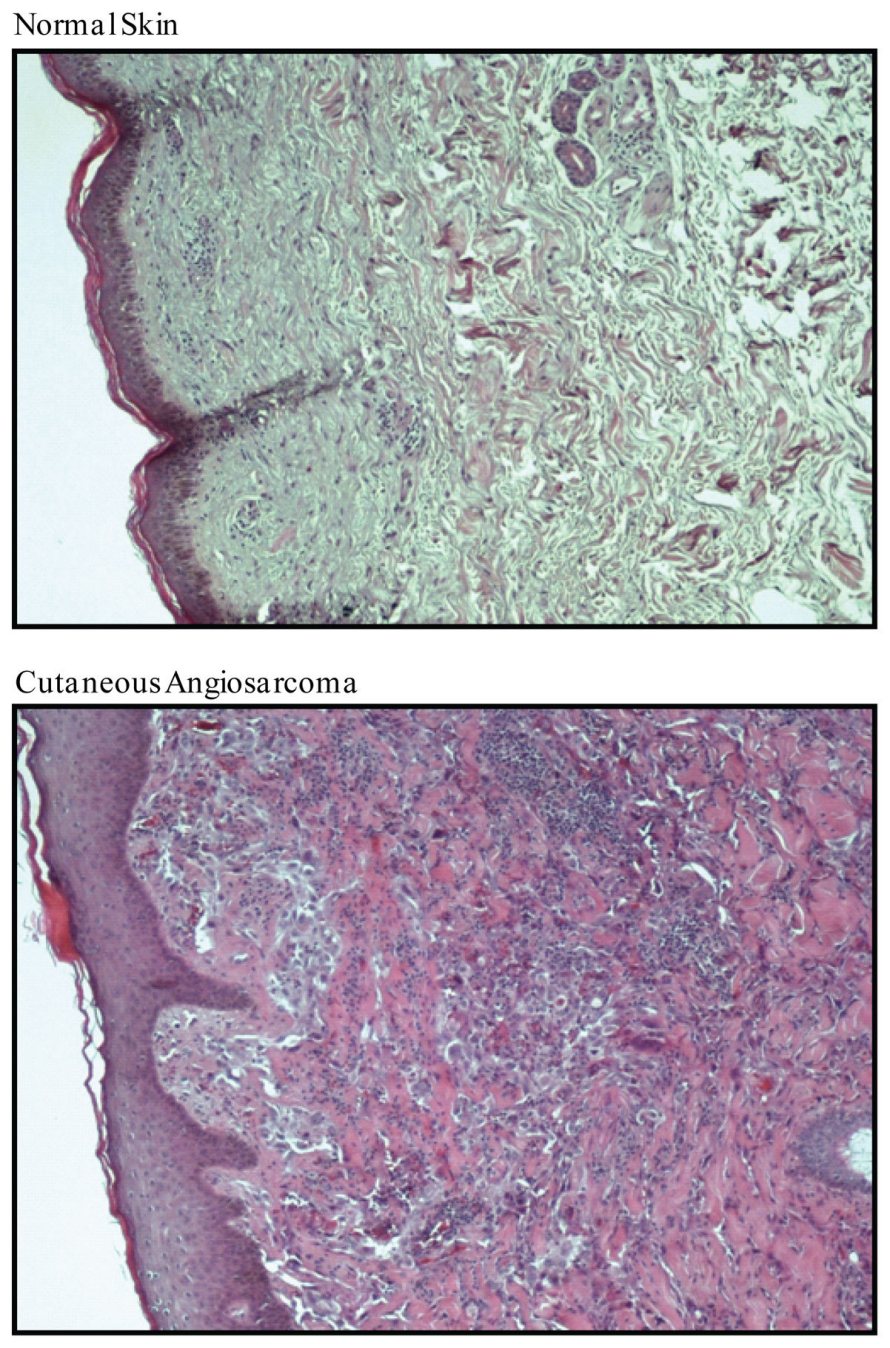
Histological comparison of cutaneous angiosarcoma to normal skin. Hematoxylin and eosin staining of neonatal foreskin and cutaneous angiosarcoma. Normal skin is characterized by highly consistent external epithelium (epidermis) and underlying connective tissue (dermis). Angiosarcomas are highly malignant tumors composed of rapidly overproliferating and aggressively infiltrating aberrant vascular cells.

Angiosarcomas, which represent approximately 2–4% of all sarcomas, are malignant neoplasms characterized by quickly proliferating, extensively infiltrating cells derived from the vascular system (Figure 1). These tumors can be divided into two classifications based on their tissue of origin-lymphangiosarcomas (which display aberrant lymphangiogenesis [i.e., overgrowth of newly formed lymph vessels]) and hemangiosarcomas (which display aberrant angiogenesis [i.e., overgrowth of newly formed blood vessels). Most angiosarcomas rapidly become metastatic because their vascular origin permits tumor dissemination without the need for initial recruitment of new blood vessels (as is a rate limiting requirement for all other solid tumors) [1–3]. Unlike epithelioid sarcomas which initially disseminate to regional lymph nodes, angiosarcomas generally metastasize directly to the lungs via the vascular system [4,5]. Due to their rapid dissemination and aggression, the median survival rate of patients with this tumor is very short-generally less than 6 months.

Angiosarcomas manifest in several different locations throughout the body with approximately 50% of the cases occurring in the head and neck region. Human soft-tissue angiosarcomas occur irrespective of the patient’s age whereas bone angiosarcomas occur in the individuals from the age of 25 and above. Like many vascular tumors, gender plays a significant role in the development of these tumors, with dermal angiosarcomas occurring more frequently in males than in females (ratio of 2:1) [6] and head and neck angiosarcomas affecting more females than males (69% and 38%, respectively) [7]. Aside from age and gender, several risk factors are associated with the development of angiosarcomas. Hepatic angiosarcoma, which is the most common sarcoma of the liver, is largely linked to toxic exposure to chemicals such as vinyl chloride, thorium dioxide, or arsenic. While primary angiosarcomas of the breast occur sporadically (0.04% of breast tumors) and usually arise in the 3^rd^ and 4^th^ decade of life [8], secondary angiosarcomas can occur in 0.1 to 0.3% of patients following breast conservation therapy combined with radiation therapy [9]. Secondary angiosarcomas are generally found in older women (usually late 60’s) who have previously undergone treatment for breast cancer and can be divided into two categories-lymphedema-associated cutaneous angiosarcoma and post irradiation angiosarcoma. Lymphedema-associated cutaneous angiosarcoma develops on the chest wall cavity and lymphedematous limbs following mastectomy and axillary lymph node dissection. The incidence of these tumors has decreased following increased use of breast conserving therapy. In contrast, post irradiation angiosarcomas generally affect the dermis or parenchyma of the breast tissue in the area previously treated by radiation. This incidence of this form of angiosarcoma has dramatically increased following breast conservation following tumor treatment [9,10].

Current treatment of angiosarcomas typically involves surgery, radiation, and neoadjuvant and/or adjuvant chemotherapy with doxorubicin or taxanes. Yet even following aggressive therapy, patient outcome is often very poor, with five year survival rates for angiosarcomas at less than 30% [11]. Multiple randomized studies failed to show a survival benefit from chemotherapy in patients with angiosarcomas, and while radiotherapy results in 80% local control of angiosarcomas, irradiation does not improve patient survival with metastatic tumors [12]. Several phase II trials have investigated the therapeutic efficacy of novel anti-angiogenic drugs such as Bevacizumab, Sunitinib, and Sorafenib against angiosarcomas [13,14]. Unfortunately, even with these molecular targeting therapeutics, a minimal to absent response was observed in these patients.

In this review, we will critically analyze recent advances in our understanding of angiosarcomas, specifically dealing with transcriptional signatures associated with aberrant angiogenesis as well as MYC amplifications in secondary angiosarcomas. Furthermore, we will identify specific areas that should be further developed to gain a better understanding of the molecular mechanisms contributing to the progression of this tumor and identify potential treatment options that should be tested to increase patient survival.

## Transcriptional Signatures of Angiosarcomas

Though angiosarcomas commonly contain mutations seen in many cancers (p53 [15], Ras [16], *BRCA* [17], and PTEN [18]), given their unique vascular nature, it is prudent to identify mutations or signaling aberrations unique to this particular solid tumor so that we can exploit this characteristic as a weakness. Though heterogeneous in clinical presentation, transcriptional profiling of angiosarcomas reveals that these tumors form a tight genomic grouping distinct from all other sarcoma types [19]. The top most upregulated genes in angiosarcomas included angiogenic regulators such as *TIE1, VEGFR2, SNRK, TEK*, and *VEGFR1*, revealing that aberrant angiogenic signaling is a key feature of this sarcoma. Indeed, compared to other sarcomas, angiosarcoma tumors exhibited higher expression levels of endothelial marker/functional genes including *PECAM1, EPHA2, ANGPT2, ENDRB, PGF, FLI1, VWF* and reduced expression levels in *KIT, VEGFA*, and *VEGFB* [19]. A comprehensive miRNome analysis of a large panel of heterogeneous human sarcomas identified 79 angiosarcoma specific alterations in miRNA expression, out of which 12 miRNAs were downregulated and 67 miRNAs were upregulated [20]. Of the highly upregulated miRNAs identified, miRDB miRNA target prediction (www.mirdb.org) indicated that miR-520c-3p, miR-519a and miR-520h potentially target a number of tumor suppressors and pro-apoptotic genes. On the contrary, highly downregulated miRNAs include miR-483-5p, miR-136 and miR-335 which putatively target oncogenes, the MAPK pathway, sarcoma specific metabolism, and cell adhesion. Comparisons of gene expression changes between primary breast angiosarcomas and secondary radiation-induced breast tumors revealed a unique oxidative stress mRNA signature as a defining characteristic of secondary angiosarcomas, even when histological and pathological features were similar between the two vascular tumor categories [21]. The authors postulated that the chronic oxidative stress could be due to mitochondrial dysfunction, dysregulated lipid oxidation, DNA damage response/repair, or oxidized misfolded proteins.

## Aberrant Angiogenic Signaling in Angiosarcoma

**Figure 2:**
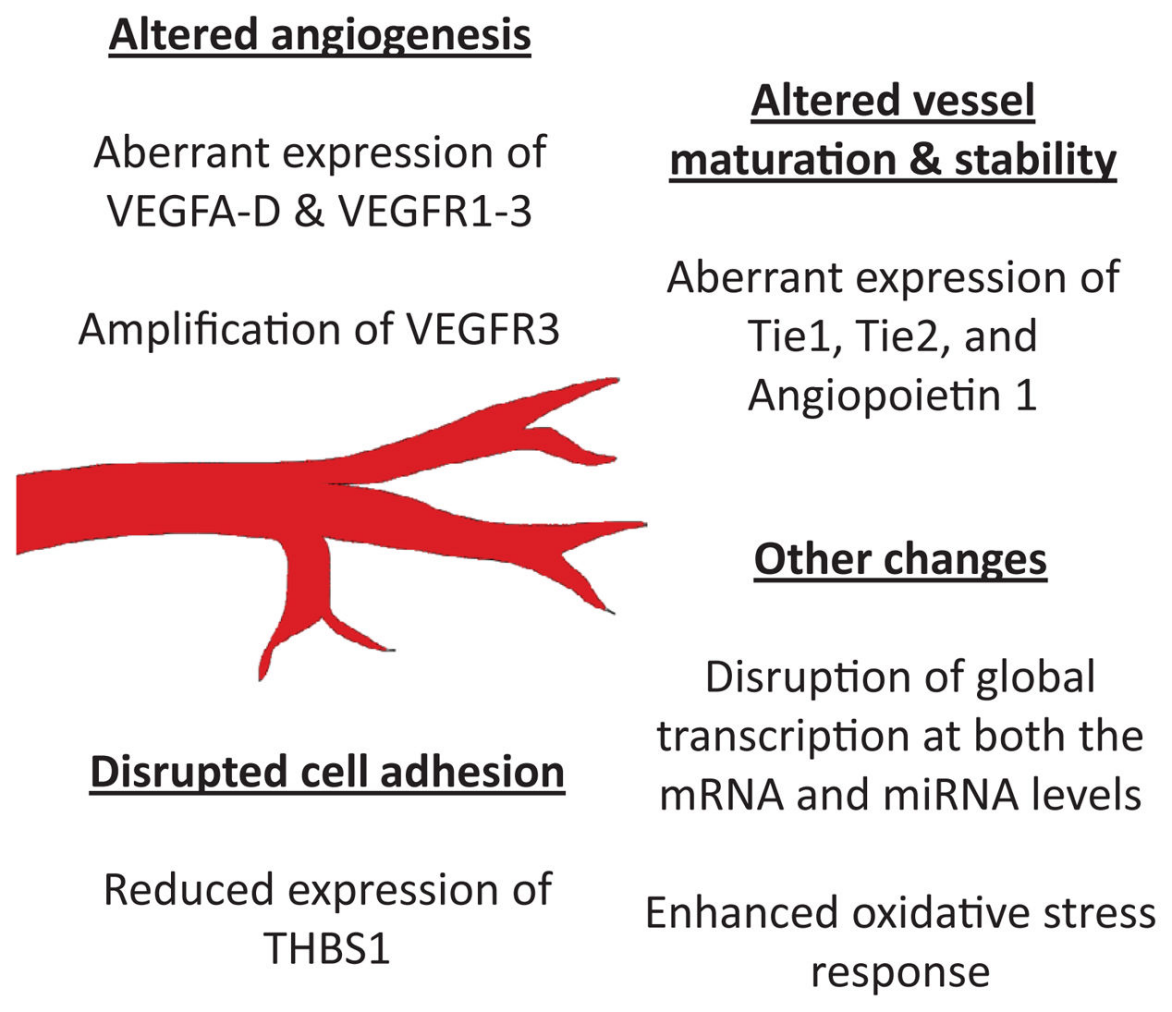
An aberrant angiogenic signature as a hallmark of angiosarcomas. Angiosarcomas are characterized by major alterations in several key angiogenic processes including disrupted expression of VEGF ligands and their cognate receptors, disrupted expression of angiopoietin ligands and their cognate Tie receptors, reduced expression of thrombospondin 1 (THBS1), and alterations in global transcriptome patterns. Moreover, secondary angiosarcomas are characterized by an enhanced oxidative stress response.

Given that angiosarcomas arise from cells of vascular origin, it seems reasonable that alterations in angiogenic signaling may be drivers in the tumor formation and progression specific to this tumor type. Moreover, it may be possible to exploit the unique vascular defects associated (Figure 2) with this tumor to our clinical advantage. In addition to high expression levels of the proliferative proteins Ki67 and cyclins A, D and E [22], angiosarcomas show remarkably variable expression in key angiogenic regulators such as VEGF-A (0–94% of angiosarcomas), VEGF-B (39% of angiosarcomas, though only tested in one report), VEGF-C (12–100% of angiosarcomas), VEGF-D (100% of angiosarcomas, though only tested in one report), *VEGFR1* (62–79% of angiosarcomas), VEGFR2 (64–94% of angiosarcomas), and VEGFR3 (79–100% of angiosarcomas) [22–29]. This data suggests that angiosarcoma progression may not only be driven by VEGF-A/VEGFR2 signaling (which dominates vascular endothelial signaling), but also by VEGF-C/VEGFR3 which is largely involved in lymphangiogenesis and maintenance of the lymphatic endothelium. Indeed, amplification of VEGFR3 occurs in 25% of secondary angiosarcomas [19,30]. As opposed to targeting the VEGF-A signaling pathway, perhaps VEGFR3 kinase blockers or neutralizing antibodies against VEGF-C may show therapeutic efficacy against specific subsets of angiosarcomas. Interestingly, the high expression of the VEGF decoy receptor *VEGFR1* appears at first paradoxical given the potent angiogenic capacity of angiosarcoma tumors. However, despite its established anti-angiogenic role, *VEGFR1* is overexpressed in a number of cancers [31,32] and is a negative prognostic factor for multiple carcinomas [33–38]. Using a canine hemangiosarcoma model which is ontogenetically related to the human disease, Tamburini et al. [39] provided strong evidence that genetic background plays an important role in predisposed susceptibility to angiosarcoma. In addition to altered expression in a disproportionate number of genes encoding transcription factors, survival factors, and pro-inflammatory regulators, the authors observed a significant enrichment of *VEGFR1* (at the mRNA and protein levels) amongst the hemangiosarcoma-prone breeds compared to less susceptible breeds. It has been postulated that enhanced expression of *VEGFR1* could be due to upregulation of Akt and ERK1/2 signaling, as these proteins have been reported to enhance its stabilization via blocking proteasomal degradation of *VEGFR1* [40]. Moreover, a novel intracellular form of *VEGFR1* has been recently discovered in breast cancer that promotes activation of the tyrosine kinase Src and enhances tumor cell invasion [41]. Similar mechanisms may exist in angiosarcoma. Point mutations in the *KDR* (VEGFR2) gene have been identified in a subset of primary and secondary angiosarcoma tumors from the breast and chest wall [19]. These mutant receptors appeared to function as constitutively active tyrosine kinases, and were susceptible to anti-angiogenic targeting by sunitinib and sorafenib. Interestingly, the authors reported low levels of VEGF-A in the angiosarcoma tumors, suggesting that angiosarcomas with low VEGF-A levels and constitutively activated VEGFR2 signaling may be better suited to targeting with tyrosine kinase inhibitors such as sunitinib or sorafenib, but not with antibody therapies such as bevacizumab [19].

In addition to VEGF signaling pathways, other angiogenic regulators are aberrantly expressed in angiosarcomas. Strong expression of *ANGPT2, TIE1*, and TEK mRNAs has been reported in cutaneous angiosarcomas [42], and Tie2 antagonists inhibit in vitro angiosarcoma cell survival and delay *in vivo* angiosarcoma tumor growth [43]. Reduced expression of thrombospondin-1 (THBS1) has been reported in MYC-amplified angiosarcomas (more on MYC amplifications in angiosarcomas below) [44], and its expression is either downregulated or lost across many cancers [45–48]. THBS1 is an extracellular glycoprotein that mediates cell-to-cell and cell-to-matrix interactions and inhibits angiogenesis via suppressing endothelial migration, proliferation, and survival [49,50]. Finally, hypoxia and subsequent HIF1-alpha protein stability has been suggested to contribute to angiosarcoma tumor progression. While sporadic cutaneous angiosarcomas have been shown to lack HIF1-alpha expression [51], other angiosarcoma subtypes (such as primary breast angiosarcoma and retroperitoneal angiosarcoma) are positive for its expression [25,52]. Indeed, using a chemically induced angiosarcoma tumor model, Laifenfield et al. [53] demonstrated that local tumor hypoxia in combination with macrophage activation and inflammation are initiating events for the formation of angiosarcomas. Hypoxia within the angiosarcoma tumor maintains genetic instability by suppressing *BRCA*1 and MLH1 activity, resulting in inhibition of DNA mismatch repair and homology specific dependent repair pathways [53–55]. As *BRCA* mutations have been associated with hereditary predisposition to angiosarcoma [56], the combination of carcinogen-mediated DNA mutations, tissue hypoxia, and hereditary aberrations in DNA repair may play a significant role in determining the incidence of angiosarcomas.

## MYC Amplification in Angiosarcoma

Benign atypical vascular lesions are common occurrences following radiation therapy and/or chronic lymphedema, and it is often difficult to differentiate between radiation induced benign vascular issues and secondary angiosarcomas due to overlapping clinical and microscopic features. Moreover, the prognosis of radiation-induced secondary angiosarcomas is significantly worse than found in sporadic angiosarcoma tumors, therefore identification of genetic biomarkers that could easily classify these groupings of vascular disorders could assist clinicians in employing the appropriate treatment option for each patient. It has been reported that the most frequent recurrent genetic alterations in secondary angiosarcomas include amplifications on chromosome 8q.24.21 (50%), 10p12.33 (33%), and 5q35.3 (11%) [57,58]. The 8q24.21 region contains oncogenes including MYC and amplification of this region is observed in several late-stage/aggressive cancers. Comparably, amplification of 10p12.33 is seen mainly in breast cancer while over-amplified 5q35.3 occurs in breast cancer, colon cancer, osteosarcoma, renal cell carcinoma, and squamous cell lung cancer. Analysis of 28 primary and 33 secondary angiosarcomas revealed that MYC amplification on chromosome 8q24.21 was found exclusively in 55% of angiosarcomas secondary to radiation or chronic lymphedema, but not in primary angiosarcomas [57]. In two other studies, the authors demonstrated that MYC amplification occurred in 100% of secondary angiosarcomas, but was absent in all cases of atypical vascular lesions [30,59]. A large scale study of 83 radiation induced sarcomas and 192 sporadic sarcomas indicated that MYC amplification was a distinguishing characteristic in radiation induced angiosarcomas, undifferentiated pleomorphic sarcomas, and leiomyosarcomas; however, the authors present significant evidence to suggest that angiosarcomas were unique amongst other sarcomas in that MYC amplifications were particularly frequent and at high levels in angiosarcomas, while other radiation induced sarcomas displayed low level MYC amplifications [60]. With sharp contrast to the previously mentioned studies Italiano et al. [61] reported data indicating that MYC is amplified in the majority of secondary angiosarcomas (67%) but is also amplified in a subset of primary angiosarcomas (50%). Taken together, these data indicate that MYC amplification is enhanced in radiation induced angiosarcomas, but may be an important mediator of primary angiosarcomas as well.

**Figure 3:**
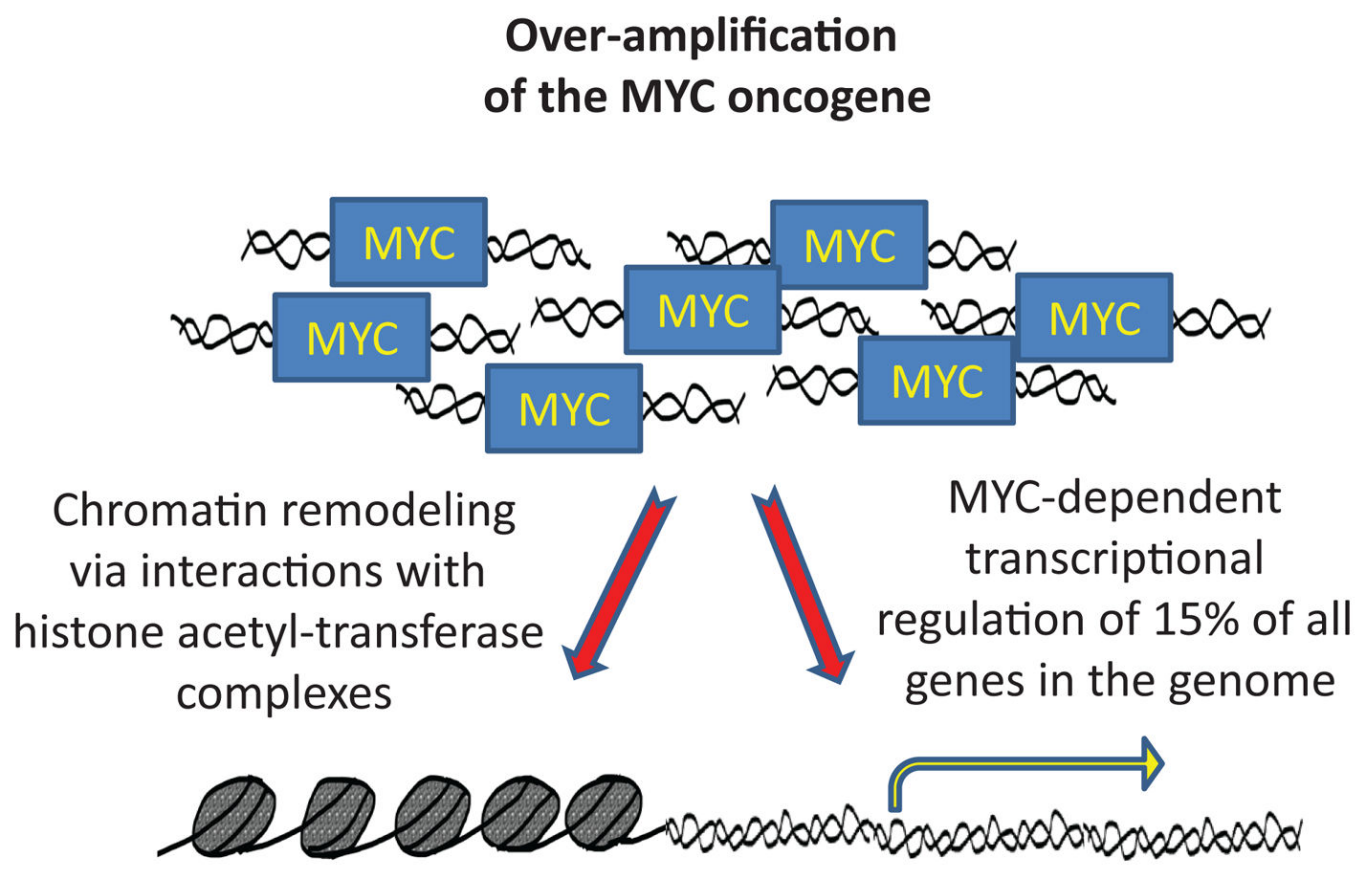
MYC amplification as a hallmark of angiosarcomas. MYC amplifications are particularly frequent and at high levels in angiosarcomas, while other sarcomas display relatively lower levels of MYC amplifications. Increased MYC activity promotes cell survival, proliferation, and plasticity via its activity as a transcription factor and through modulation of chromatin remodeling.

What are the effects of MYC amplification in angiosarcomas (Figure 3)? MYC is an oncogenic transcription factor perhaps correctly referred to as the “oncogene from hell [62]” which regulates the expression of approximately 15% of all genes to promote cell survival, proliferation, and plasticity [63]. MYC belongs to the basic helix-loop-helix (bHLHZ) superfamily of transcription factors and uniquely exerts its effects through both transcriptional activity and modulation of chromatin architecture via regulating histone acetyl-transferases [64,65]. MYC is expressed at high levels in most tumors, and several tumor types also contain translocations, amplifications, and mutations in key MYC regulators [66]. Aberrant MYC signaling in cancers is associated with poor clinical outcomes, increased rates of metastasis, tumor recurrence, and patient mortality. It is believed that elevated MYC signaling amplifies the activity of all expressed genes in a tumor cell, thus sending the cell’s gene expression program into overdrive and dramatically overwhelming any inhibitory factors that might prevent cell proliferation [67]. Moreover, MYC is upregulated during hypoxia via a HIF-dependent mechanism [68] and plays a major role in regulating physiological and tumor angiogenesis and inflammation [69]. Almost nothing has been reported regarding the contribution of MYC to the angiosarcoma transcriptome, however a substantial upregulation of the miR-17-92 cluster (a miRNA polycistron also known as oncomir-1) occurs in radiation induced secondary angiosarcomas harboring MYC amplifications compared to secondary angiosarcomas without the amplification [61]. Upregulation of this miRNA cluster occurs across diverse cancers and its expression promotes tumor cell invasion and proliferation [70–76]. As mentioned above, THBS1 expression is lost in MYC amplified angiosarcomas. Interestingly, members of the miR17-92 cluster target THBS1 directly [61], demonstrating one mechanism by which MYC amplification may induce an aberrant angiogenic phenotype in angiosarcomas. As standard chemotherapy and even novel anti-angiogenic treatments have largely failed patients stricken with angiosarcoma, strategies for exploiting MYC dependency in angiosarcoma tumors are an attractive disease-specific goal. Unfortunately, despite intense research efforts to inhibit MYC activity, this protein has thus far remained an elusive cancer therapy target.

## Conclusions and Future Directions

Limited data exists evaluating the molecular mechanisms controlling angiosarcomas, however a wealth of recent publications have shown that key features of angiosarcomas include aberrant angiogenic signaling, increased oxidative stress, and MYC amplification. Future studies should focus on classifying heterogenous angiosarcomas based on unique molecular profiles so that therapeutic treatments can be personalized specific to the genetic and signaling aberrations unique to each individual tumor.
